# Optimization of Therapy and the Risk of Probiotic Use during Antibiotherapy in Septic Critically Ill Patients: A Narrative Review

**DOI:** 10.3390/medicina59030478

**Published:** 2023-02-28

**Authors:** Maria Ioana Onofrei, Cristina Mihaela Ghiciuc, Catalina Mihaela Luca, Paraschiva Postolache, Cristina Sapaniuc, Georgiana Enache Leonte, Florin Manuel Rosu

**Affiliations:** 1 Clinic of Infectious Diseases, “Sf. Parascheva” Clinical Hospital of Infectious Diseases, 700116 Iasi, Romania; 2 Department of Infectious Diseases, Grigore T. Popa University of Medicine and Pharmacy of Iasi, 16 Universitatii Street, 700115 Iasi, Romania; 3 Pharmacology, Clinical Pharmacology and Algeziology, Department of Morpho-Functional Sciences II, Faculty of Medicine, Grigore T. Popa University of Medicine and Pharmacy of Iasi, 16 Universitatii Street, 700115 Iasi, Romania; 4 Department of Medicine I—Pulmonary Rehabilitation Clinic, Grigore T. Popa University of Medicine and Pharmacy of Iasi, 16 Universitatii Street, 700115 Iasi, Romania

**Keywords:** septic patients, critically ill, therapeutic drug monitoring, pharmacokinetics, pharmacodynamic, PK/PD relationship, probiotics, synbiotics, septic stress

## Abstract

Optimizing the entire therapeutic regimen in septic critically ill patients should be based not only on improving antibiotic use but also on optimizing the entire therapeutic regimen by considering possible drug–drug or drug–nutrient interactions. The aim of this narrative review is to provide a comprehensive overview on recent advances to optimize the therapeutic regimen in septic critically ill patients based on a pharmacokinetics and pharmacodynamic approach. Studies on recent advances on TDM-guided drug therapy optimization based on PK and/or PD results were included. Studies on patients <18 years old or with classical TDM-guided therapy were excluded. New approaches in TDM-guided therapy in septic critically ill patients based on PK and/or PD parameters are presented for cefiderocol, carbapenems, combinations beta-lactams/beta-lactamase inhibitors (piperacillin/tazobactam, ceftolozane/tazobactam, ceftazidime/avibactam), plazomicin, oxazolidinones and polymyxins. Increased midazolam toxicity in combination with fluconazole, nephrotoxic synergism between furosemide and aminoglycosides, life-threatening hypoglycemia after fluoroquinolone and insulin, prolonged muscle weakness and/or paralysis after neuromuscular blocking agents and high-dose corticosteroids combinations are of interest in critically ill patients. In the real-world practice, the use of probiotics with antibiotics is common; even data about the risk and benefits of probiotics are currently spares and inconclusive. According to current legislation, probiotic use does not require safety monitoring, but there are reports of endocarditis, meningitis, peritonitis, or pneumonia associated with probiotics in critically ill patients. In addition, probiotics are associated with risk of the spread of antimicrobial resistance. The TDM-guided method ensures a true optimization of antibiotic therapy, and particular efforts should be applied globally. In addition, multidrug and drug–nutrient interactions in critically ill patients may increase the likelihood of adverse events and risk of death; therefore, the PK and PD particularities of the critically ill patient require a multidisciplinary approach in which knowledge of clinical pharmacology is essential.

## 1. Introduction

Most critically ill patients are susceptible to serious infections with multidrug-resistant (MDR) agents due to exposure to several factors: invasive procedures (central venous catheter, mechanical ventilation, vesical catheterization), which may be sources of biofilm-producing MDR bacteria; history of colonization or infection with MDR pathogens; immunosuppression and disruption of defense mechanisms (e.g., cough reflex); trauma, surgery, treatment (corticoids, sedation, stress ulcer prophylaxis); or disease-related immunosuppression [1]. Studies published during the COVID-19 pandemic found an elevated prevalence of 30–50% of superinfections with MDR bacteria (e.g., *Enterococcus faecium, Staphylococcus aureus, Acinetobacter baumannii*, carbapenem-resistant *Enterobacterales*, and *Pseudomonas aeruginosa*) in critically ill COVID-19 patients admitted to intensive care units (ICUs), and their severity was directly correlated to corticoids and mechanical ventilation [2]. The EUROBACT I trial, which included 1156 critically ill patients from 24 ICUs in 24 countries, revealed an MDR incidence of 47.8%, including 20.5% extended drug-resistant and 0.5% pan-drug-resistant patterns [3]. A lower but significant prevalence of MDR agents (14.1%) was reported in 652 critically ill patients included in the DEFINE trial [4]. Therefore, ICUs are regarded as centers of the emergence, amplification, and spread of MDR infections [1]. The harmless symbiotic population of bacteria was found to be significantly reduced in the detriment of pathogens with MDR potential, such as *Enterococcus* spp. or *Clostridium* spp., in hospitalized patients compared with healthy people [5,6]. The therapeutic management of critically ill patients is a challenge not only because of MDR pathogens but also due to the multiple organ dysfunction syndrome (MODS). MODS in critically ill patients includes a life-threatening intestinal dysfunction caused by the dysregulated host response to infection [7].

A bidirectional relationship was seen between xenobiotics and organ disfunction in critically ill patients. On the other hand, dysbiosis is a factor that modifies drug disposition. Moreover, the administration of antibiotics (mainly broad spectrum), antacids, sedatives, opioids, catecholamines, neuromuscular blockade, enteral/parenteral feeding, and even supine position may have devastating effects with disruption of gut microbiota and risk of selection of resistant bacteria [5].

Inappropriate antibiotic therapy may seriously impact the microenvironment, favoring the development of MDR bacterial or fungal superinfections. For this reason, antibiotic stewardship programs (ASP) were developed to limit the antimicrobial resistance phenomenon, following the same steps: (1) rapid identification of patients requiring antibiotic therapy; (2) initiation of empirical antibiotic treatment; (3) optimization and individualization of treatment; and (4) de-escalation of antibiotic therapy [8]. According to the Antimicrobial Stewardship, Therapeutic Drug Monitoring and Early Appropriate infection Management in European ICUs (A-TEAMICU) survey, which included 812 participants from 71 countries, ASPs are available in 63% of ICUs [9]. In the precise medicine, therapeutic drug monitoring (TDM) is a tool to control the drugs’ toxicity and to increase effectiveness with positive pharmacoeconomic impact. This method is essential in critically ill patients in absence of the development of new antimicrobial classes in parallel with an antimicrobial resistance (AMR) rate highly increased [10]. However, optimizing the therapeutic regimen in septic critically ill patients should be based not only on improving antibiotic consumption but also on optimizing the entire therapeutic regimen by considering possible drug–drug or drug–nutrient interactions. One of the most used classes of xenobiotics used in association with antibiotics is probiotics; even the current recommendations are against the use of probiotics for the prevention of *Clostridioides difficile* infection [11].

In this context, the aim is to review recent approaches used to optimize the therapeutic regimen in septic critically ill patients based on pharmacokinetics and pharmacodynamic characteristics.

## 2. Materials and Methods

**Figure 1:**
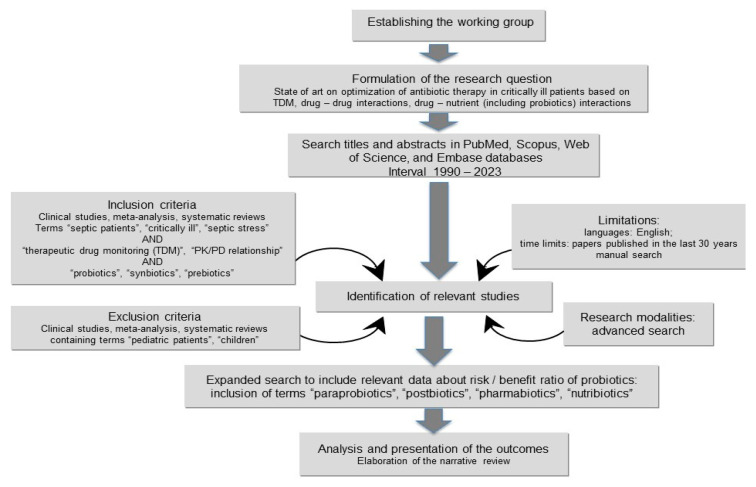
Flow chart of the literature search.

Our aim was to realize a narrative synthesis on recent pharmacokinetics and pharmacodynamic-based advances to optimize the therapeutic regimen in septic critically ill patients; therefore, we followed the basic principles of a systematic review. The literature was searched independently by two authors (MIO and CMG), up to January 2023, in PubMed, Scopus, Web of Science, and Embase databases. We used the terms “septic patients”, “critically ill”, “therapeutic drug monitoring (TDM)”, “pharmacokinetics (PK)”, “pharmacodynamic (PD)”, “PK/PD relationship”, “probiotics”, “synbiotics”, “prebiotics”, “paraprobiotics”, “postbiotics”, “pharmabiotics”, “nutribiotics” and “septic stress” in different combinations. Title and abstract were screened, and we selected relevant articles to be read in their full form. Through the snowballing search, we screened the references of the selected articles for additional articles to be included (Figure 1). Including criteria were studies on recent advances on TDM-guided drug therapy optimization based on PK and/or PD results in septic critically ill patients. Excluded criteria were studies including patients under the age of 18 years old or studies with classical TDM-guided optimization of drug therapy in septic critically ill patients. We organized our narrative review in one section with new approaches in TDM-guided recommendations in septic critically ill patients based on PK and/or PD parameters and two sections on aspects of high interest in critically ill patients (antibiotic–drug interactions and the risk of probiotic use).

## 3. Results

### 3.1. TDM-Guided Recommendations in Septic Critically Ill Patients

Antibiotic treatment should be adjusted based on the PD properties, the pathogens’ susceptibilities (as assessed by their minimal inhibitory concentrations (MICs)) and PK characteristics of antibiotics in the critically ill host. In clinical practice, MIC is the most used PD parameter. Knowledge of MIC is the foundation for selecting antimicrobial therapy against bacteria and fungi and helps to guide dosing needs in critically ill patients [12]. Its main limitation is that knowing MIC does not account for the individual host defenses and does not provide sufficient information on the patterns of exposure to antimicrobial agents for an optimal therapeutic response [13]. In addition, bacterial status (tolerance or persistence), bacterial inoculum size, or antibiotic concentrations may influence their antimicrobial activity [14].

Pathological changes in critically ill patients in the volume of drug distribution, protein binding, and clearance led to a high intra- and inter-individual PK variability that significantly alters exposure to antibiotics with the risk of suboptimal doses or increased risk of toxicity. Depending on their PK/PD characteristics (Table 1), the PK parameters (e.g., C_max_, C_min_ or AUC) should be assessed in relation to MIC to establish the effectiveness and safety of antibiotic therapy [15,16,17]. Recently, a new concept of the maximum tolerable dose (MTD) for beta-lactams in critically ill patients was proposed as the highest dose deemed safe for the patient, which has the goal of maximizing the kill of bacteria and minimizing the risk of antimicrobial resistance and toxicity, but there are a lack of data on the association of beta-lactam antibiotic levels and markers of toxicity [18].

For this reason, model-informed precision dosing (MIPD), such as populational PK, PK/PD models in combination with TDM, Bayesian algorithms, and Monte Carlo simulations, are important tools for the individualization of treatment in critically ill patients. TDM quantifies drug concentrations (in plasma) using validated bioanalytical methods, while drug exposure may be directly correlated with the therapeutic target for PD response (MIC) or estimated through Bayesian methods. Dosing nomograms, clinician-based predictions, or dosing software (e.g., BestDose^®^, Antibiotics kinetics^®^, MwPharm++^®^, TDMx^®^, etc.) allow for further individual dosage adjustment [19,20,21,22]. TDM is already successfully implemented on a large scale; A-TEAMICU reported that TDM was used in 61% of ICUs surveilled [9].

In 2020, experts from many worldwide associations on antibiotic treatment revised the TDM guidelines. Accordingly, TDM is routinely recommended for various beta-lactams, aminoglycosides, linezolid, teicoplanin, or vancomycin in critically ill patients [23].

#### 3.1.1. Beta-Lactams TDM-Guided Recommendations

Beta-lactams have a time-dependent bactericidal activity. The maximum bactericidal activity of beta-lactams is considered to occur when free fractions are maintained at least four times above the MIC over the entire dosing interval (i.e., 100% *f*T > 4xMIC) with an increase up to 8xMIC in critically ill septic patients, while minimum plasma concentration (C_min_) estimates antibiotics toxicity (e.g., the cut-off value for C_min_—361 mg/L for penicillins or 20 mg/L for cephalosporins) [24,25].

Cefiderocol is a novel siderophore cephalosporin approved for infections caused by multidrug-resistant aerobic Gram-negative organisms in adults with limited treatment options and *f*C_min_/MIC ratio is considered the optimal PK/PD parameter. In a case series of 13 patients with extended drug-resistant *Acinetobacter baumanii*, microbiological failure was reported in 80% of patients with suboptimal *f*C_min_/MIC (<1) compared with 29% of those with optimal or quasi-optimal *f*C_min_/MIC ratio (≥4, and respectively 1–4) [26].

For carbapenems, the optimal clinical results in critically ill patients are 100%ft > MIC and 100%ft > 4xMIC or target through concentration >4–8xMIC, while a C_min_ of 64.2 mg/L is potentially neurotoxic [27]. In patients with normal renal function, the usual recommended doses are often subtherapeutic and do not reach the target concentrations. In a meta-analysis including 35 studies, we noted that meropenem should be administered in doses up to 6 g /day (every 6 h, 8 h or 12 h) or by continuous infusion or prolonged infusion (up to 3 h) to achieve the PK/PD target [28,29].

#### 3.1.2. Beta-Lactam and Beta-Lactamase Inhibitor Combinations TDM-Guided Recommendations

As many authors have highlighted, the widely used antipseudomonal piperacillin/tazobactam had a wide PK variability in ICU, and varied target concentrations could not be achieved. TDM-guided dose modifications dramatically improved therapeutic exposure and defined target concentrations of 100 mg/L and 361 mg/L for nephrotoxicity and neurotoxicity, respectively [27,30]. Nevertheless, the recently published TARGET international study findings are not promising. After evaluating 244 patients, the authors found that TDM-guided piperacillin/tazobactam medication had no beneficial impact on critically ill patients [31].

Ceftazidime/avibactam possesses a high activity against many carbapenem-resistant *Enterobacterales* and *Pseudomonas aeruginosa* with a therapeutic target of 24–30 mg/L in plasma and 8–10 mg/L at the site of infection; dosage adjustment based on TDM led to therapeutic failure in 1% of cases [32].

Ceftolozane/tazobactam was recently approved for treating intraabdominal, renal, and lower respiratory infection (including ventilation-acquired pneumonia) caused by multiresistant *Enterobacterales*, *Pseudomonas aeruginosa* or *Haemophilus influenzae*. In 40 patients with multi- or extended drug-resistant *P. aeruginosa* infections, TDM-recommended dose reductions were applied in 84.2% of cases receiving prolonged infusion and allowed the achievement of 100%ƒT ≥ MIC target even at lower doses [33,34].

#### 3.1.3. Aminoglycosides TDM-Guided Recommendations

Aminoglycosides are concentration-dependent antimicrobials with known toxicity and very high PK variability in critically ill patients. TDM methods are already implemented at a large scale.

Plazomicin is a new aminoglycoside antibiotic effective against *Enterobacterales* (including carbapenemase-producing bacteria), *Pseudomonas aeruginosa*, and *Staphylococcus* spp., including methicillin-resistant strains. In patients with normal renal function, the recommended dosage of plazomicin is 15 mg/kg per day; TDM is recommended in patients with a creatinine clearance <90 mL/minute to maintain plasma trough concentrations below 3 g/mL [35].

#### 3.1.4. Oxazolidinones TDM-Guided Recommendations

From the oxazolidinones group, Linezolid is extensively used in ICUs to treat infections produced by Gram-positive multiresistant cocci. It has a narrow therapeutic index and a high PK variability in critically ill patients; the usual recommended doses of 1.2 g/day often led to subtherapeutic levels. The ratio of the area under the drug plasma concentration–time curve over 24 h to the MIC (AUC/MIC) or percentage time above the MIC (%T > MIC) are predictors of the therapeutic response, and concentrations in the range of 2–8 mcg/mL appear to define the optimal window for acute bacterial infections, while a cut-off value >8 mg/L is an indicator for thrombocytopenia. Thus, TDM based on PK models or continuous infusion over 6 h instead of intermittent infusion is recommended for the optimization of Linezolid treatment to achieve the target AUC/MIC [17,36,37,38,39].

#### 3.1.5. Polymyxins TDM-Guided Recommendations

Even though there is not a well-defined recommendation for polymyxins (polymyxin B, colistin) monitoring, a narrow therapeutic index related to nephrotoxicity and the ratio of the area under the unbound plasma concentration–time curve over a dosing interval to minimum inhibitory concentration (*f*AUC:MIC) is considered the most predictive PK/PD parameter of colistin activity [40,41]. Many authors consider polymyxin E as the subject of TDM in ICUs as an adaptive feedback control because of the following: (1) CMS and colistin prodrug concentrations are not correlated, and (2) in critically ill patients, kidney function and renal replacement therapy dramatically impact the PK of prodrug CMS and colistin with the risk of under or overdosing [42,43,44].

However, these are only a few examples of TDM–guided recommendations for antibiotics usually used in critically ill patients, and many other methods are published in the literature.

### 3.2. Antibiotic–Drug Interactions of High Interest in Critically Ill Patients

Changes in PK and PD in critically ill patients are of concern not just for antibiotics but also for many other ICU-used drugs (including sedatives, anesthetics, or cardiovascular medication). Due to their polypharmacy, these patients may be exposed to potentially harmful PK or PD drug–drug interactions. Of interest could be reactions, such as increased midazolam toxicity in combination with fluconazole, nephrotoxic synergism between furosemide and aminoglycosides, possible life-threatening hypoglycemia after fluoroquinolone and insulin combination, prolonged muscle weakness including myopathy, and/or paralysis after the combination of neuromuscular blocking agents and high doses of corticosteroids, but the examples are much more numerous [45,46]. Due to these considerations, the therapy must be individualized based on the patient’s features (such as obesity for lipophilic medications, changes in drug disposition such as hypoalbuminemia, renal and hepatic function), medical history, and concomitant medication. Numerous applications, such as Lexicomp^®^, Micromedex, Drugs.com^®^, and Stockley’s drug interactions, provide vast and constantly updated databases for optimizing drug interactions [47]. However, many of these platforms with various performances are not systematically validated and may lead to suboptimal clinicians’ decisions, particularly in critically ill patients [48].

### 3.3. The Risk of Probiotic Use in Critically Ill Patients

Probiotics are commonly used in clinical practice to combat antibiotic-associated side effects (such as *Clostridioides difficile* infection and diarrhea, even in critically ill septic patients. Yet, this combination of probiotics and antibiotics should be regarded as a “double-edged sword” where the hazards may outweigh the benefits [49].

Often marketed as natural health products, functional foods, dietary supplements or medical devices, their beneficial effects on critically ill patients have been a topic of interest for many authors only in recent years [50]. Supplements are considered either *pharmabiotics* (with a proven pharmacological role in health or disease and specific health claims) or *nutribiotics* (food, a food product, or dietary supplements that are subject to regulatory standards pertaining to food safety and nutritional recommendations) [51]. Despite the rising global usage of supplements such as probiotics, prebiotics, synbiotics, paraprobiotics and postbiotics (Table 2) in various health-promoting goods for their immunomodulatory and antibacterial activities [52], there are still major gaps in our understanding of these products and their implication for therapeutics in critically ill patients. Paraprobiotics and postbiotics are new entities derived from *Lactobacillus* species that consist of a wide range of effector molecules with similar benefits to *lactobacillus* but have the advantage of clear chemical structure and safe dose parameters [53,54]. 

Clinical studies showed the benefits of probiotics in several pathological states, such as metabolic diseases (obesity, insulin resistance syndrome, type 2 diabetes, and non-alcoholic fatty liver disease), allergic diseases (e.g., atopic dermatitis), depression, and gastrointestinal diseases (e.g., irritable bowel syndrome, gastrointestinal disorders, *Helicobacter pillory* infection, inflammatory bowel disease) [55]. *Lactobacillus* (*L. acidophilus, L. rhamnosus, L. casei*, etc.), *Bifidobacterium* spp., and yeasts (e.g., *Saccharomyces boulardii*) are the most extensively assessed probiotic strains introduced in international guidelines for the treatment of diarrhea (including antibiotic-associated diarrhea and traveler’s diarrhea) or *C. difficile* infections, but their use for these indications is not agreed by all associations [11,56,57]. However, Lerner et al. (2019) cited the findings of a recent study indicating that “post-antibiotic gut mucosal microbiome reconstruction is impaired by probiotics” [58].

The optimal probiotic for critically ill patients could not be ascertained, although probiotics had some beneficial effects in clinical studies (e.g., reduced antibiotic use and incidence of *C. difficile* infections), with effects dependent on various variables (e.g., probiotic strain or disease state, age, lifestyle, diet). ICU patients were assessed for various outcomes, including the occurrence of ventilator-associated pneumonia (VAP) nosocomial infections, hospitalization length, and death. Almost all published studies have shown that probiotics reduce the incidence of VAP but had no significant impact on hospitalization duration, mortality, duration of mechanical ventilation, and even the incidence of diarrhea (Table 3) [59,60,61,62,63,64,65,66,67,68]. In the PROSPECT study, probiotics were not superior to placebo in terms of the incidence of antibiotic-associated diarrhea (52.4% vs. 50.0%, *p* = 0.57) or *C. difficile* infections (2.4% vs. 2.1%, respectively, *p* = 0.60) among 2653 critically ill patients in the largest multicenter international placebo-controlled trial [69]. The variety of strains investigated, dosages, timing, length of therapy, and contradictory findings preclude a clear conclusion regarding the effectiveness of probiotics in critically ill patients, and more well-planned multicenter trials are necessary.

According to current legislation, probiotics used as supplements do not require safety monitoring as medicinal products. However, the Food and Drug Administration and European Food Safety Authority periodically update evaluations and recommendations regarding the safety of food products, including probiotics [70]. The most often reported side effects of probiotics are gastrointestinal (constipation/diarrhea, bloating, gastrointestinal ischemia, thirst, or taste changes), skin (rash, acne), excessive immune activation, and metabolic problems (e.g., obesity, D-lactic acidosis, or metabolic acidosis). Several cases of bacteremia caused by *L. rhamnosus* in immunocompromised pediatric patients and fungemia caused by *Saccharomyces* spp. in immunocompromised individuals were reported. There are also reports of endocarditis, meningitis, endometritis, peritonitis, and pneumonia associated with probiotics [71,72]. Recently, Israeli and American researchers released the findings of a five-year study involving more than 2,000 critically ill patients. They concluded that ICU patients had an elevated risk of probiotic-associated bacteremia [73].

Understanding the role of microbiota on antibiotic resistance has been significantly enhanced by the discovery of the resistome [73]. In non-pathogenic bacteria, both intrinsic non-transmissible resistance and acquired resistance due to spontaneous gene mutations (non-transmissible) or horizontal gene transfer (transformation, phage-associated transduction, or transfer of mutant genomes via integrons and transposons) pose a threat. *Lactobacilli*, the most prevalent strain in probiotics, feature a gene reservoir that contains transferrable resistance genes. *Bacillus subtilis* strains also provide the potential for horizontal gene transfer to other close bacteria [74].

Although the current guidelines offer recommendations only for certain strains that have been studied, there are other marketed probiotics which contain other bacterial strains (e.g., *Enterococcus* spp. or *Clostridium butyricum*) which are not included in the EFSA’s Qualified presumption of safety (QPS) due to the identification of toxins in some strains (e.g., *Clostridium* spp.) or to the risk of becoming opportunistic pathogens with MDR risk (e.g., *Enterococcus* spp.) [75,76]. Such probiotics must be used with great care only after a good analysis of the risk/benefit ratio in ICU patients.

### 3.4. Multidisciplinary Approach Therapy Optimization in Septic Critically Ill Patients

There are multiple challenges in critically ill patients obtaining clinical and microbiological cures. These patients are more susceptible to being infected with multidrug-resistant or pan-drug-resistant pathogens, with limited therapeutic options and the necessity of choosing the rescue antibiotics (such as polymyxins or Linezolid) associated with a high degree of toxicity [36,42]. In addition, the substantial intra- or inter-individual variation in PK characteristics often leads to suboptimal or toxic therapeutic levels. In this context, the implementation of the Italian TDM-guided expert clinical pharmacologist advice (ECPA) program allowed dose optimization for antibiotics used for MDR infections, such as piperacillin–tazobactam, carbapenems, but also Linezolid [77].

**Figure 2:**
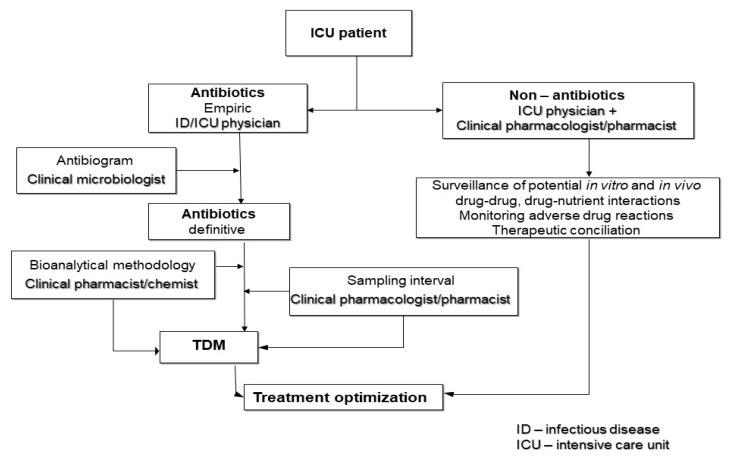
Decision-making steps of optimization of therapy in septic critically ill patients [78,79].

Each step for the management of critically ill patients is highly relevant, but the optimization of antibiotic treatment applying principles of 3D (*right drug—right dose—right duration*) in conjunction with in vivo and in vitro drug interactions and even drug–nutrient interaction is essential and often needs well-trained interdisciplinary teams in which the clinical pharmacologist should play an essential role (Figure 2) [78,79].

## 4. Discussions

AMR is rightly considered a silent pandemic since the annual mortality rate associated with drug-resistant infections has an average of 4.95 million deaths worldwide [80]. Without an appropriate measure, this may increase to 10 million deaths annually by 2050 [81]. ASP, as a measure within the One Health transdisciplinary action, decreased the consumption of antibiotics by up to 28% [82]. Moreover, implementing ASP using a multidisciplinary approach for PK/PD analysis reduced the rate of MDR in ICU-acquired infections by almost 9% [83]. TDM has been designed since 1960 and has seen widespread expansion with the development of clinical pharmacology departments. Although it has disadvantages, such as the need to have well-trained multidisciplinary teams or the costs related to the development of sensitive and accurate bioanalytical methods, it is now widely used for a variety of diseases (rheumatic, cardiovascular, inflammatory, neuropsychiatric, oncological) for a variety of molecules, including biological therapy [84,85,86]. As a result, every effort should be made to develop protocols for TDM, particularly in countries with a known AMR rate and/or high antibiotic consumption [87].

In addition, the optimization of antibiotic therapy should consider drug–drug interactions (DDIs). In a large, multicenter observational study, 48.5% of ICU patients were found to have DDIs. The most frequent DDIs reported were between QT-prolonging drugs and between NSAIDs and other serotoninergic agents (e.g., selective serotonin reuptake inhibitors or Linezolid) [88].

However, not all drug interactions are well defined even though they may cause harm. Considering the note of Morrow et al. (2012), “in an era of increasing antibiotic resistance among pathogens and limited new antibiotics in the research pipeline, probiotics offer clinicians promise,” the consumption of probiotics worldwide is extremely high despite the little knowledge of these products [50]. Since only several probiotics are authorized as medical devices and the majority are supplements, their approval process differs from that of medicines and varies from country to country. This can lead to discrepancies in individual requirements, labels, and warnings about safety. Most results regarding the effectiveness of probiotics are inconclusive or even contradictory, and adverse reactions can be serious in critically ill patients (they can even produce acidosis).

However, despite regulatory differences on international or regional levels, regulators worldwide have two common concepts in mind: to avoid misleading the consumer and to ensure the product’s safety. Therefore, in vitro and in vivo studies, including appropriately designed, adequately powered experimental trials meeting CONSORT criteria, are required to evaluate the product’s efficacy and safety [89]. Until the implementation of these requests, doctors must be aware of the entire treatment of critically ill patients, including the composition of the chosen probiotic and their possible negative impact, to reduce the risks of medical errors. In addition, investigator-initiated studies should be encouraged to obtain information about probiotics from real-world settings.

## 5. Conclusions

The TDM-guided method ensures a true optimization of antibiotic therapy, and particular efforts should be made to apply it globally. In addition, it is recognized that multidrug interactions in critically ill patients increase the likelihood of adverse events and risk of death.

Even though pharmacologically active medications are well-known, probiotics frequently used in association with antibiotics have only lately attracted interest, and recent data on the safety and interactions of probiotics are equivocal or contradictory. Therefore, the PK and PD particularities of the critically ill patient require a multidisciplinary approach in which knowledge of clinical pharmacology is essential in the context of the polypharmacy of this group of patients.

## Figures and Tables

**Table 1:** PK/PD relationship of antibiotics [16,17].

PK/PD Relationship	Antibacterial Class	PK, PD Parameters
Time-dependent	β-lactams, linezolid, lincosamides, macrolides	*f*T > MIC
Concentration-dependent	aminoglycosides, fluoroquinolones, daptomycin	*f*Cmax/MIC
Co-dependent (concentration-dependent with time-dependence)	fluoroquinolones, tigecycline, linezolid, glycopeptides, macrolides, colistin	*f*AUC24/MIC

Abbreviations. fT > MIC: time that free serum concentration above minimum inhibitory concentration; fCmax/MIC: maximum free serum concentration divided by MIC; fAUC24/MIC: area under the curve of free serum concentration divided by MIC.

**Table 2:** Examples of supplements [53,54,55].

Product	Strain
Probiotics	*Lactobacillus (e.g., L. acidophilus, L. rhamnosus, L. reuteri, L. bulgaricus, L. plantarum, L. casei, L. lactis, etc.)*, *Bifidobacterium (B. bifidum, B. longum, B. breve, B. infantis, B. lactis, B. thermophilum*, etc.)*, Saccharomyces* spp. (*S. cerevisiae, S. boulardii*), lactic acid bacteria (LAB) including *Lactococcus*, *Lactobacillus, Streptococcus*, and *Enterococcus* or Clostridium spp. cluster IV
Prebiotics	galactooligosaccharides (GOS), fructooligosaccharides (FOS), xylooligosaccharides (XOS), isomaltooligosaccharides (IMO), inulin, lactulose, lactosucrose, lactitol
Synbiotics	*L. rhamnosus* + inulin, *Lactobacillus + Bifidobacterium + Enterococcus* + FOS, *Saccharomyces boulardii + Lactobacillus sporogenes* + FOS)
Paraprobiotics	peptidoglycans, teichoic acid, cell-wall polysaccharides, cell surface-associated proteins, proteinaceous filaments
Postbiotics	Exopolysaccharides, short chain fatty acids, enzymes, bacterial lysates, etc.

**Table 3:** Meta-analysis and systematic reviews of the effectiveness of probiotics, prebiotics, and synbiotics in critically ill patients [59,60,61,62,63,64,65,66,67,68].

Author	Method	No. Studies/ No. Patients	Results	Conclusion/Limitations
Li et al., 2022 [59]	meta-analysis	31 RCT 8339 patients	- nutritional supplementation with probiotics and synbiotics reduced the risk of VAP and nosocomial infections, respectively. - prebiotics are the most effective in preventing diarrhea (possible antibiotic-induced).	- probiotics may offer an advantage in critically ill patients, - recommendation limited by the low quality of analyzed studies.
Naseri et al., 2022 [60]	umbrella review	20 RCT	- probiotics may reduce the rate of ventilator-associated pneumonia, nosocomial pneumonia, the overall infection rate, duration of mechanical ventilation, and antibiotic use. - no significant association between probiotics and mortality, length of hospitalization, incidence of diarrhea.	- not enough evidence to support the routine use. - further well-designed multicenter trials needed.
Sun et al., 2022 [61]	meta-analysis	23 RCT 5543 patients	- probiotics reduced the risk of VAP. - no effects on 28-/90-day mortality, nosocomial infections, bacteremia, diarrhea.	- prophylactic probiotics might be a preventive method for VAP. - further large, high-quality RCTs needed.
Weng et al., 2017 [62]	meta-analysis with trial sequential analysis	13 studies 1969 patients	- reduced incidence of VAP. - no significant difference in mortality (28-/90-day and overall—ICU or hospital) - no significant difference in length of ICU/ hospital stays. - no significant difference in duration of mechanical ventilation and diarrhea.	- quality of trials relatively low. - insufficient information. - further trials needed.
Cheema et al., 2022 [63]	systematic review and meta-analysis	18 RCTs 4893 patients	- probiotics may reduce the incidence of VAP (not significant). - probiotics reduced the length of ICU stay and the duration of antibiotic use.	- low quality of trials. - further large-scale trials are needed.
Su et al., 2020 [64]	meta-analysis	14 studies 1975 patients	- significant reduction in VAP incidence among all studies but not among the double-blinded studies. - no statistically significant differences for ICU mortality, ICU stay, duration of mechanical, occurrence of diarrhea.	- additional large-scale and multicenter RCT needed.
Bo et al., 2014 [65]	Cochrane review	8 RCTs 1083 patients.	- probiotics decreased the incidence of VAP (low-quality evidence). - uncertain results for ICU mortality, in-hospital mortality, length of ICU stays, duration of mechanical ventilation, antibiotic use.	- low quality of evidence. - not enough evidence for conclusions.
Manzanares et al., 2016 [66]	systematic review and meta-analysis	30 RCT 2972 patients	- significant reduction in infections (p = 0.09). - significant reduction in the incidence VAP. - no effect on mortality, length of hospitalization or diarrhea. - subgroup analysis: probiotics more efficient than synbiotics.	- not sufficient evidence for final strong recommendation. - probiotics should be considered to improve outcome in critically ill patients. - further trials needed.
Barraud et al., 2013 [67]	meta-analysis	13 trials 1439 patients	- probiotics reduced the incidence of ICU-acquired pneumonia and length of stay in ICU. - probiotics did not significantly reduce ICU or hospital mortality. - probiotics use did not shorten duration of mechanical ventilation or hospital length of stay.	- heterogenicity between study treatments.
Wang et al., 2022 [68]	systematic review and meta-analysis	25 RCT 5049 patients	- reduction in incidence of VAP significant only in patients receiving synbiotics and not significant in those receiving only probiotics. - no significant difference in mortality, length of ICU stays, diarrhea.	- heterogenicity between study treatments.

## Data Availability

Not applicable.
